# Direct Oral Anticoagulants in Patients with Atrial Fibrillation and Bioprosthetic Valves—A Systematic Review and Meta-analysis

**DOI:** 10.19102/icrm.2021.121202

**Published:** 2021-12-15

**Authors:** Mohammed Ruzieh, Deborah L. Wolbrette, Gerald V. Naccarelli

**Affiliations:** ^1^Division of Cardiovascular Medicine, University of Florida College of Medicine, Gainesville, FL, USA; ^2^Division of Cardiology, Penn State Heart and Vascular Institute, Penn State College of Medicine, Hershey, PA, USA

**Keywords:** Atrial fibrillation, bioprosthetic valves, bleeding, DOACs, stroke

## Abstract

Oral anticoagulation is recommended for patients with atrial fibrillation and an elevated stroke risk. Direct oral anticoagulants (DOACs) are generally preferred over vitamin K antagonists. Nonetheless, there controversy persists regarding whether DOACs should be used in patients with atrial fibrillation and bioprosthetic valves. Therefore, we conducted this systematic review and meta-analysis to assess the safety and efficacy of DOACs compared to warfarin in this patient population. We performed a systematic search of the MEDLINE and PubMed Central databases for relevant articles. The incidence rate and risk ratio (RR) of all-cause mortality, cardiovascular mortality, ischemic stroke/systemic thromboembolism, hemorrhagic stroke/intracranial bleeding, major bleeding, and minor bleeding were calculated. A total of eight studies were included, including 5,300 patients (stratified as 1,638 patients in the DOAC arm and 3,662 patients in the warfarin arm). There was no significant difference in the rate of stroke/systemic thromboembolism [RR: 0.85; 95% confidence interval (CI): 0.43–1.69], all-cause mortality (RR: 0.77; 95% CI: 0.53–1.11), or cardiovascular death (RR: 0.81; 95% CI: 0.40–1.63) between DOACs and warfarin. Major bleeding and hemorrhagic stroke/intracranial bleeding were similar between both treatment arms (RR: 0.61; 95% CI: 0.35–1.06 and RR: 0.27; 95% CI: 0.06–1.13, respectively). In conclusion, DOACs are safe and effective in patients with atrial fibrillation and bioprosthetic valves. Future large-scale randomized studies are warranted to confirm this observation.

## Introduction

Atrial fibrillation is the most common cardiac arrhythmia in the elderly,^1^ and it increases the risk of stroke and systemic embolism by about fivefold.^2^ Anticoagulation using vitamin K antagonists (warfarin)^3^ or direct oral anticoagulants (DOACs)^4–7^ is recommended in patients with atrial fibrillation and an elevated stroke risk.^8^ Recent guidelines recommend DOACs as first-line therapy over warfarin in patients with atrial fibrillation. Therefore, the rate of DOAC use has steadily increased over the past decade.^9^

The presence of mechanical valve or moderate-to-severe mitral stenosis is a contraindication to DOACs based on the results of the Randomized, Phase II Study to Evaluate the Safety and Pharmacokinetics of Oral Dabigatran Etexilate in Patients After Heart Valve Replacement (RE-ALIGN)^10^ and the exclusion of these patients from the randomized trials of DOACs versus warfarin.^4–7^ On the other hand, patients with bioprosthetic valves were represented in small numbers in some of the trials of DOACs in patients with atrial fibrillation.^11,12^ A recent randomized clinical trial of rivaroxaban versus warfarin in patients with atrial fibrillation and bioprosthetic mitral valves found similar rates of death and ischemic stroke between both groups.^13^

There is still controversy regarding DOACs should be used in patients with atrial fibrillation and bioprosthetic valves. Therefore, we conducted this systematic review and meta-analysis to address this question and assess the safety and efficacy of DOACs in this population.

## Methods

### Data source and search strategy

We followed the guidelines of the Preferred Reporting Items for Systematic Reviews and Meta-Analyses statement for reporting systematic reviews. We performed a systematic search of MEDLINE and PubMed Central databases from January 1, 2000, to March 15, 2021, for relevant articles. The search syntax for DOACs in patients with atrial fibrillation and bioprosthetic valves was as follows: [direct anticoagulants (title/abstract) OR novel anticoagulants (title/abstract) OR dabigatran (title/abstract) OR rivaroxaban (title/abstract) OR apixaban (title/abstract) OR edoxaban (title/abstract)] AND [atrial fibrillation (title/abstract)] AND [valvular (title/abstract) OR bioprosthetic (title/abstract) OR valve replacement (title/abstract) OR tissue valve (title/abstract)] AND [clinical study (publication type) OR clinical trial (publication type) OR controlled clinical trial (publication type) OR observational study (publication type) OR pragmatic clinical trial (publication type) OR randomized controlled trial (publication type)]. In addition, our search was supplemented by a manual review of the references from the articles retrieved.

### Study selections

Two authors (M. R. and G.V.N.) independently screened the articles for inclusion and extracted baseline characteristics and outcome data. Disagreement was resolved by consensus. We included all study designs of DOACs versus warfarin in patients with atrial fibrillation and bioprosthetic valves.

### Data extraction and outcomes

We performed standardized extraction of the following study characteristics: mean age, sex, study design, type of atrial fibrillation, location of the bioprosthetic valve, follow-up time, comorbid conditions, and antiplatelet use. Endpoints of all-cause death, cardiovascular death, valve thrombosis, stroke, systemic thromboembolism, or bleeding events were collected.

### Quality of trials

The risk-of-bias tool developed by the Cochrane Collaboration was used to assess the risk of bias in randomized clinical trials, while ROBINS-I, a tool for assessing the risk of bias in nonrandomized studies of interventions, was used to assess the risk of bias in observational studies.

### Data analysis

We calculated the risk ratio (RR) of each outcome using the Mantel–Haenszel random-effects model. Examination of heterogeneity was performed using I^2^. Randomized and nonrandomized studies were combined to increase the power of the analysis and to assess heterogeneity among studies. Sensitivity analysis was performed by individually excluding studies from the model. Analysis was performed using Review Manager Web (RevMan Web; The Cochrane Collaboration, London, England). A two-sided p-value of less than 0.05 was considered statistically significant.

## Results

**Figure 1:**
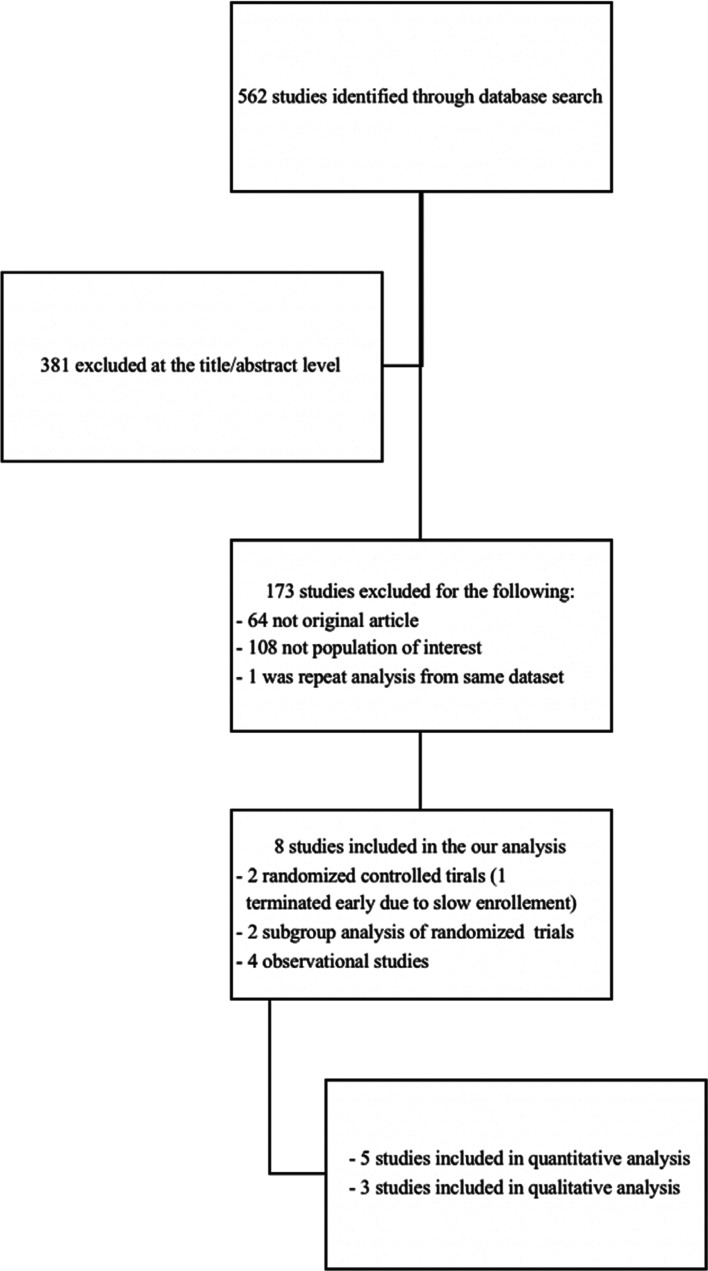
Preferred Reporting Items for Systematic Reviews and Meta-analyses diagram of included articles.

**Figure 2:**
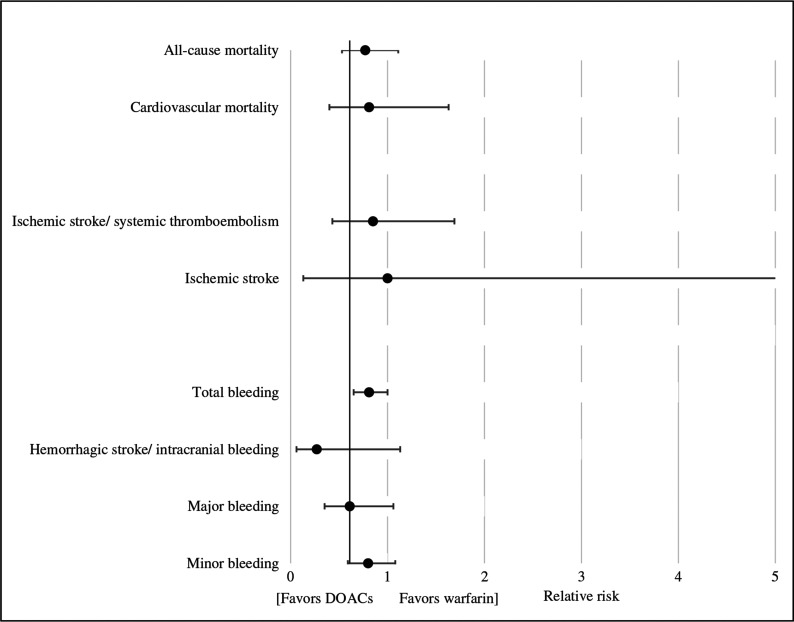
Relative risk study of outcomes for DOACs versus warfarin. DOACs: direct oral anticoagulants.

A total of eight studies met our inclusion criteria and hence were included in this systematic review and meta-analysis (two randomized controlled trials,^13,14^ two subgroup analysis or randomized trials,^11,12^ and four observational studies^15–18^ **(Figure 1)**. Data from five studies were available for pooled analysis.^11,13–15,17^ There were 1,638 patients in the DOAC arm and 3,662 patients in the warfarin arm **(Table 1)**. All bioprosthetic valves were either mitral or aortic with no representation from patients with bioprosthetic tricuspid or pulmonic valves. The study outcomes are summarized in **Table 2 and Figure 2**.

### Risk of bias of assessment

The risk of bias was variable among the included studies **(Table 3)**. Two randomized trials^13,14^ were designed to assess whether DOACs were non-inferior to warfarin in patients with atrial fibrillation and bioprosthetic valves. Both of them were open-label studies, and the outcome assessment was blinded in only one.^13^ The Dabigatran Versus Warfarin After Mitral and/or Aortic Bioprosthesis Replacement and Atrial Fibrillation Postoperatively (DAWA) pilot study was stopped early due to slow recruitment.^14^ For nonrandomized studies, the major biases were classifications of the intervention and deviation from the intended intervention as studies did not adjust for prescription refills, compliance to the drugs, time in the therapeutic range for warfarin, and crossover between study groups.

### Outcomes

**Figure 3:**
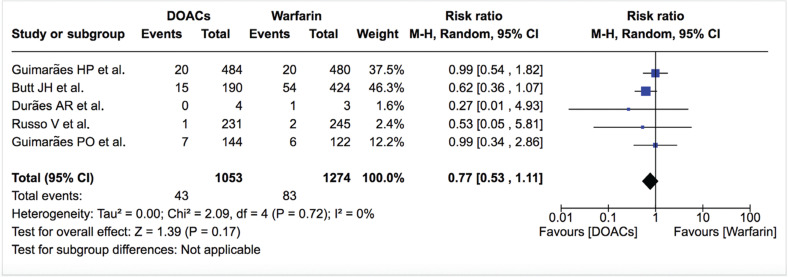
Relative risk of all-cause mortality for DOACs vs. warfarin. “Total” represents patient-years.

**Figure 4:**
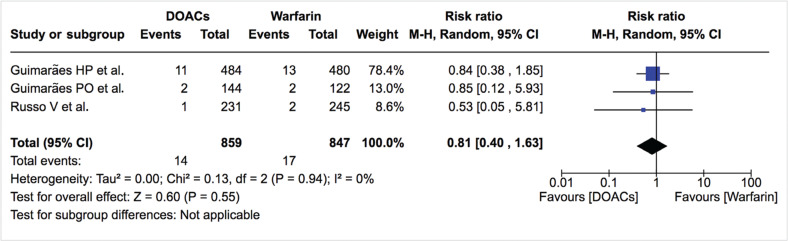
Relative risk of cardiovascular mortality for DOACs vs. warfarin. “Total” represents patient-years.

***Mortality.*** Mortality data were available from six studies. The rates of all-cause mortality and cardiovascular mortality were similar between DOACs and warfarin [4.1%/year vs. 6.5%/year; RR: 0.77; 95% confidence interval (CI): 0.53–1.11, and 1.6%/year vs. 2.0%/year; RR: 0.81; 95% CI: 0.40–1.63, respectively] **(Table 2, Figures 3 and 4)**. The treatment effect was similar among studies (I^2^: 0%) and did not change on sensitivity analysis. All-cause mortality was also similar between DOACs and warfarin in the study by Duan et al.^16^ (4.0%/year vs. 5.3%/year; hazard ratio: 0.87; 95% CI: 0.72–1.05).

**Figure 5:**
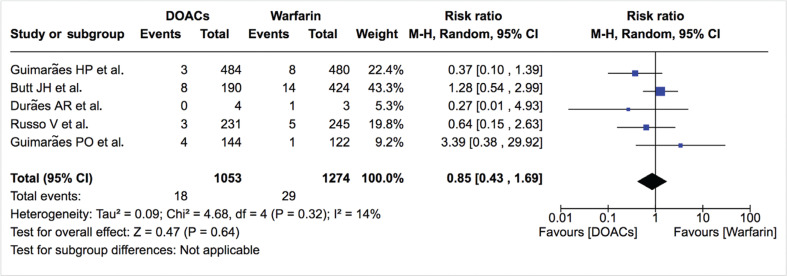
Relative risk of ischemic stroke/systemic thromboembolism for DOACs vs. warfarin. “Total” represents patient-years.

**Figure 6:**
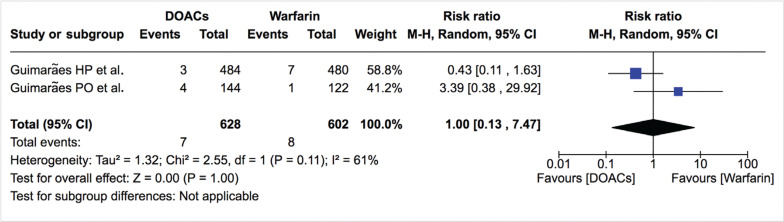
Relative risk of ischemic stroke for DOACs vs. warfarin. “Total” represents patient-years.

***Thromboembolic disease.*** The rate of ischemic stroke/systemic thromboembolism was not significantly different between DOACs and warfarin (1.7%/year vs. 2.3%/year; RR: 0.85; 95% CI: 0.43–1.69) **(Figure 5)**. Heterogeneity was low (I^2^: 14%). The rate of ischemic stroke was also similar between DOACs and warfarin (1.1%/year vs. 1.3%/year; RR: 1.00; 95% CI: 0.13–7.47) **(Table 2, Figure 6)**. For studies not included in the quantitative analysis, both Duan et al.^16^ and Carnicelli et al.^12^ reported a similar rate of stroke between both treatment arms (2.9%/year vs. 2.4%/year; p > 0.05 and 1.2% vs. 1.7%; p > 0.05, respectively). Di Biase et al.^18^ reported no thromboembolic events in the DOAC or warfarin group at 12 months.

**Figure 7:**
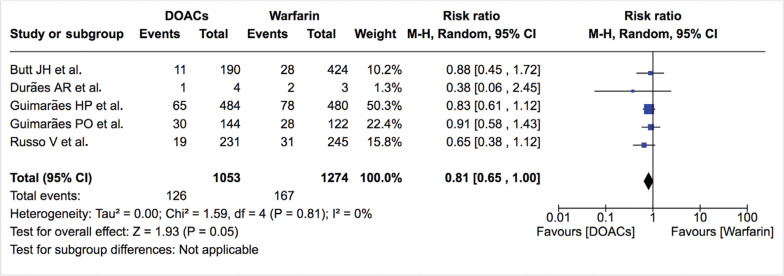
Relative risk of total bleeding events for DOACs vs. warfarin (all studies). “Total” represents patient-years.

**Figure 8:**
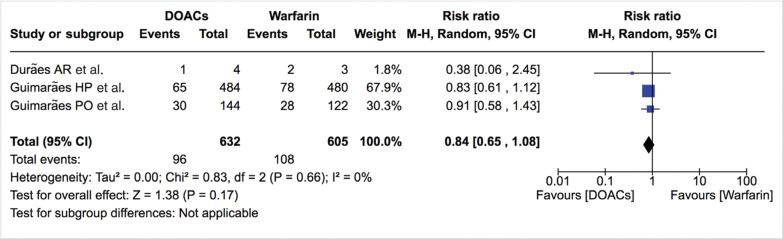
Relative risk of total bleeding events for DOACs vs. warfarin (randomized studies). “Total” represents patient-years.

**Figure 9:**
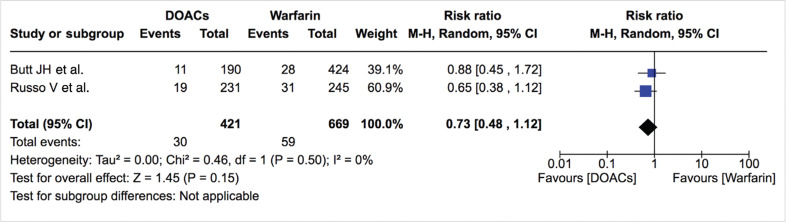
Relative risk of total bleeding events for DOACs vs. warfarin (observational studies). “Total” represents patient-years.

**Figure 10:**
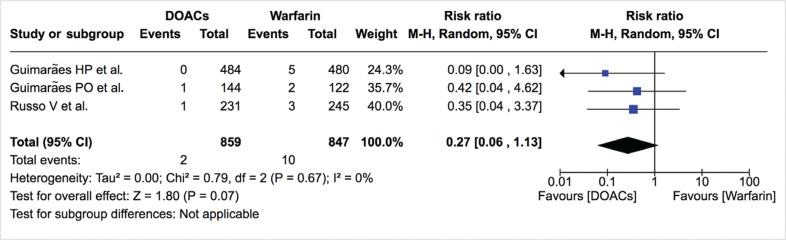
Relative risk of hemorrhagic stroke/intracranial bleeding for DOACs vs. warfarin. “Total” represents patient-years.

**Figure 11:**
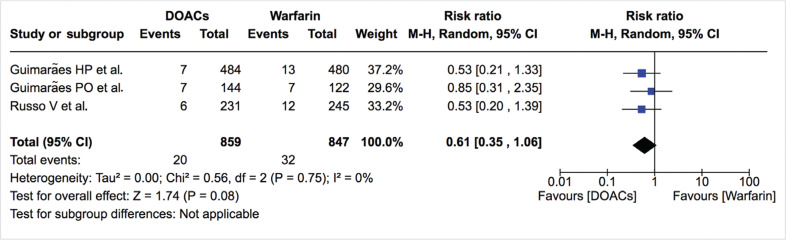
Relative risk of major bleeding for DOACs vs. warfarin. “Total” represents patient-years.

**Figure 12:**
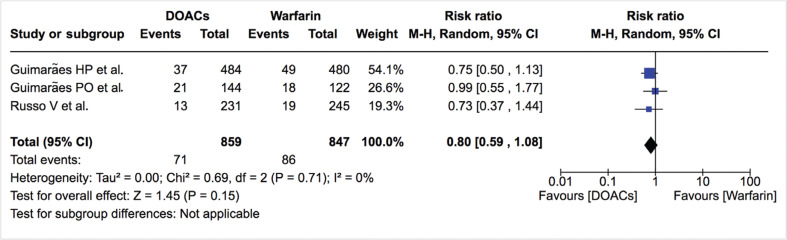
Relative risk of minor bleeding for DOACs vs. warfarin. “Total” represents patient-years.

***Bleeding events.*** The incidence of total bleeding events was similar between DOACs and warfarin (12.0% vs. 13.0%; RR: 0.81; 95% CI: 0.65–1.00) **(Figures 7–9)**. On the other hand, fewer patients in the DOAC arm had hemorrhagic stroke/intracranial bleeding or major bleeding compared to those in the warfarin arm (0.2% vs. 1.2%; RR: 0.27; 95% CI: 0.06–1.13 and 2.3% vs. 3.8%; RR: 0.61; 95% CI: 0.35–1.06, respectively) **(Table 2, Figures 10 and 11)**. Nonetheless, this did not reach statistical significance due to being underpowered for the comparison. Heterogeneity was low for all endpoints (I^2^ = 0%). Minor bleeding was similar between both groups **(Figure 12)**. The lower rate of hemorrhagic stroke/intracranial bleeding with DOACs was also seen in the study by Duan et al.^16^ (0.6% vs. 1.2%) and Carnicelli et al.^12^ (0.0% vs. 3.5%).

## Discussion

In this systematic review and meta-analysis of the use of DOACs in patients with atrial fibrillation and bioprosthetic heart valves, the rate of all-cause mortality, cardiovascular death, and thromboembolic events was similar among patients who received DOACs and their counterparts who received warfarin. Heterogeneity was low and the treatment effect was consistent among all studies included. Total bleeding events were similar between DOACs and warfarin. Nonetheless, rates of intracranial bleeding/hemorrhagic stroke and major bleeding were lower with DOACs, although this study was underpowered to confirm the statistical significance for this finding.

Warfarin has a narrow therapeutic range, requires frequent monitoring, and has numerous drug and dietary interactions. Conversely, DOACs (dabigatran, rivaroxaban, apixaban, and edoxaban) are given at a fixed dose, do not require routine monitoring, and have fewer drug–drug interactions. The findings of this study support switching patients with atrial fibrillation and bioprosthetic valves from warfarin to a DOAC.

The authors recognize several limitations to this study. First, only two randomized controlled trials^13,14^ were available for analysis, of which one was terminated early due to slow enrollment.^14^ The Rivaroxaban for Valvular Heart Disease and Atrial Fibrillation (RIVER) trial^13^ included patients with bioprosthetic mitral valves only, and it is unknown whether these results hold true for patients with bioprosthetic valves in other positions. However, current evidence suggests a similar rate of stroke in patients with bioprosthetic mitral and aortic valves,^19^ and there is no pathophysiological reason to suggest that patients with aortic bioprosthesis would be less responsive to treatment with DOACs than patients with mitral bioprosthesis. Second, the subgroup analysis of Apixaban for Reduction in Stroke and Other Thromboembolic Events in Atrial Fibrillation (ARISTOTLE) and Global Study to Assess the Safety and Effectiveness of Edoxaban (DU-176b) vs. Standard Practice of Dosing with Warfarin in Patients with Atrial Fibrillation (ENGAGE AF-TIMI 48) trials had a small number of patients, 156 and 191 patients, respectively. Finally, the observational studies did not assess for medication adherence or time in therapeutic range for warfarin, which could have influenced the results of these studies. Nonetheless, the rates of thromboembolic events, total bleeding events, and major bleeding events were not significantly different than what is seen in landmark trials of DOACs in patients with atrial fibrillation, which validates the results of this analysis.^4–7^

In conclusion, DOACs are safe and effective in patients with atrial fibrillation and bioprosthetic valves. More prospective trial data are needed to confirm these findings.

## Figures and Tables

**Table 1:** Baseline Characteristics of Patients in the Included Studies

Study	Anticoagulation Group (Number of Patients)	Age, Mean ± SD	Female Sex, n (%)	CHA_2_DS_2_-VASc Score, Mean ± SD	Location of Bioprosthetic Valve	Antiplatelets, n (%)
Guimarães et al.^11^	Warfarin (69)	74*	27 (39.1%)	51 (73.9%) with score ≥ 2 points**	Mitral in 26 (16.7%) and aortic in 73 (46.8%); valve repair in rest	27 (39.1%)
	Apixaban (87)	72*	34 (39.1%)	56 (63.4%) with score ≥ 2 points**		26 (29.9%)
Carnicelli et al.^12^	Warfarin (70)	75*	70 (36.6%)	3 ± 1 points**	Mitral in 131 (68.6%) and aortic in 60 (31.4%)	Aspiring in 65 (34.0%)
	Edoxaban (121)					
Guimarães et al.^13^	Warfarin (505)	59.2 ± 11.8	296 (58.6%)	2.5 ± 1.3 points	Mitral in all	64 (12.7%)
	Rivaroxaban (500)	59.4 ± 2.4	311 (62.2%)	2.7 ± 1.5 points		75 (15%)
Durães et al.^14^	Warfarin (12)	45.7 ± 6.0	7 (58.3%)	NA	Mitral in 9 (75.0%)	NA
	Dabigatran (15)	48.8 ± 10.4	10 (66.7%)		Mitral in 11 (73.3%)	
Russo et al.^15^	Warfarin (130)	65.7 ± 8.9	58 (44.5%)	3.2 ± 1.2 points	Mitral in 68 (52.3%) and aortic in 62 (47.7%)	9 (6.9%)
	DOACs (130)	66.1 ± 8.5	56 (43.1%)	3.1 ± 1.1 points	Mitral in 64 (49.2%) and aortic in 66 (50.8%)	8 (6.2%)
Duan et al.^16^	Warfarin (2,233)	1,292 (86.4%) ≥ 65 years	871 (39.0%)	2,124 (95.1%) with score ≥ 2 points	Mitral in 839 (37.6%) and aortic in 1,392 (62.3%)	654 (29.3%)
	DOACs (439)	386 (87.9%) ≥ 65 years	181 (41.2%)	424 (96.6%) with score ≥ 2 points	Mitral in 104 (23.7%) and aortic in 332 (75.6%)	173 (39.4%)
Butt et al.^17^	Warfarin (516)	82*	239 (46.3%)	4.9 ± 1.3 points	TAVR in all	395 (76.5%)
	DOACs (219)	83*	101 (46.1%)	5.0 ± 1.4 points		182 (83.1%)
Di Biase et al.^18^	Warfarin (127)	63.0 ± 10.9	43 (33.9%)	2.74 ± 1.3 points	Mitral in 54 (42.5%) and aortic in 73 (57.5%)	NA
	DOACs (127)	63.0 ± 10.9	43 (33.9%)	2.71 ± 1.3 points	Mitral in 52 (40.9%) and aortic in 75 (59.1%)	

DOACs: direct oral anticoagulants; NA: not available; SD: standard deviation; TAVR: transcatheter aortic valve replacement.

*Median.

**CHADS_2_.

**Table 2:** Summary of Findings

Outcomes	Anticipated Absolute Effects (95% CI)		Relative Risk (95% CI)	No. of Participants (Studies)	Quality of Evidence
	Incidence Rate per Year with DOACs	Incidence Rate per Year with Warfarin			
Overall mortality	4 per 100	7 per 100	0.77 (0.53–1.11)	2,183 (5)	Moderate
Cardiovascular mortality	2 per 100	2 per 100	0.81 (0.40–1.63)	1,421 (3)	Moderate
Ischemic stroke/systemic thromboembolism	2 per 100	2 per 100	0.85 (0.43–1.69)	2,183 (5)	Moderate
Ischemic stroke	1 per 100	1 per 100	1.00 (0.13–7.47)	1,161 (2)	Moderate
Total bleeding	12 per 100	13 per 100	0.81 (0.65–1.00)	2,183 (5)	Moderate
Hemorrhagic stroke/intracranial bleeding	0 per 100	1 per 100	0.27 (0.06–1.13)	1,421 (3)	Moderate
Major bleeding	2 per 100	4 per 100	0.61 (0.35–1.06)	1,421 (3)	Moderate
Minor bleeding	8 per 100	10 per 100	0.80 (0.59–1.08)	1,421 (3)	Moderate

CI: confidence interval; DOACs: direct oral anticoagulants.

**Table 3:** Bias assessment for the included studies

Randomized Controlled Trials							
Study	Random Sequence Generation (Selection Bias)	Allocation Concealment (Selection Bias)	Blinding of Participants and Personnel (Performance Bias)	Blinding of Outcome Assessment (Detection Bias)	Incomplete Outcome Data (Attrition Bias)	Selective Reporting (Reporting Bias)	Other Biases
Guimarães et al.^11^	Low	Low	Low	Low	Low	Low	Low
Carnicelli et al.^12^	Low	Low	Low	Low	Low	Low	Low
Guimarães et al.^13^	Low	Moderate	High	Low	Low	Low	Low
Durães et al.^14^	Low	Low	High	High	High	Low	Low
**Nonrandomized Studies**							
**Study**	**Bias due to Confounding**	**Bias in the Selection of Study Participants**	**Bias in the Classification of Interventions**	**Bias due to Deviations from Intended Interventions**	**Bias due to Missing Data**	**Bias in Measurement of Outcomes**	**Bias in the Selection of the Reported Results**
Russo et al.^15^	Low	Moderate	Unclear	Unclear	Low	Low	Low
Duan et al.^16^	Low	High	High	High	Low	Low	Low
Butt et al.^17^	Moderate	Low	High	High	Low	Low	Low
Di Biase et al.^18^	Moderate	Moderate	low	Unclear	Unclear	Low	Unclear
